# Diseases of the Rectum

**Published:** 1855-11

**Authors:** 


### BOOK NOTICES.

Diseases of the Rectum. By W. Bodenhamer, M. D., Second Edition Illustrated by plates, and exemplified by numerous cases: New York Published for the author, by J. S. Redfield, 1855.

We have received from D. B. Cook & Co., the above work, which is especially addressed to the non-medical reader.

The author professes to have cured upwards of seven hundred cases of fistula in ano during the last seventeen years without either the knife, the actual, or the potential cautery. We will not pretend to controvert the Doctor's statement. But if true we think his first duty should have been to publish to the profession of which he claims to be an honorable member, the principles on which his treatment is based, and the mode by which it is conducted to such a happy termination.

Among the causes of rectal disease the author mentions excessive purgation and tight lacing. The dangers from irregular habits so far as defecation is concerned, are dwelt on to some extent. The author justly remarks that "we are creatures of habit," and that there are many persons who, by their continued applications to the temple of Cloacina for relief in this respect, have finally obtained it permanently. In addition to this as a means of overcoming constipation cold water enemata are strongly recommended together with exercise, a regulation of the diet and early rising.

About forty pages are devoted to the subject of hæmorrhoids. The whole work may conveniently be divided into two portions, one of which contains much useful and interesting matter that should be known to the public, the other is made up of letters from private individuals, and newspaper puffs, *a la* the medical almanacs and humbug pamphlets of the day.

The writer intimates that he treats complete anal fistula always by the ligature peculiarly applied. The details of his method, however, are not given, but are promised in a forthcoming work on these affections designed for the profession. J.

